# Safety and feasibility of prolonged versus early laparoscopic cholecystectomy for acute cholecystitis: a single-center retrospective study

**DOI:** 10.1007/s00464-020-07643-z

**Published:** 2020-05-22

**Authors:** Xing Cheng, Ping Cheng, Peng Xu, Ping Hu, Gang Zhao, Kaixiong Tao, Guobin Wang, Xiaoming Shuai, Jinxiang Zhang

**Affiliations:** 1 Department of Emergency Surgery, Union Hospital, Tongji Medical College, Huazhong University of Science & Technology, Wuhan, 430022 Hubei People’s Republic of China; 2 Department of Gastrointestinal Surgery, Union Hospital, Tongji Medical College, Huazhong University of Science & Technology, Wuhan, 430022 Hubei People’s Republic of China

**Keywords:** 72 h of symptoms onset, Acute cholecystitis, Prolonged versus early laparoscopic cholecystectomy

## Abstract

**Background:**

Laparoscopic cholecystectomy (LC) is the standard treatment for acute cholecystitis (AC), and it should be performed within 72 h of symptoms onset if possible. In many undesired situations, LC was performed beyond the golden 72 h. However, the safety and feasibility of prolonged LC (i.e., performed more than 72 h after symptoms onset) are largely unknown, and therefore were investigated in this study.

**Methods:**

We retrospectively enrolled the adult patients who were diagnosed as AC and were treated with LC at the same admission between January 2015 and October 2018 in an emergency department of a tertiary academic medical center in China. The primary outcome was the rate and severity of adverse events, while the secondary outcomes were length of hospital stay and costs.

**Results:**

Among the 104 qualified patients, 70 (67.3%) underwent prolonged LC and 34 (32.7%) underwent early LC (< 72 h of symptom onset). There were no differences between the two groups in mortality rate (none for both), conversion rates (prolonged LC 5.4%, and early LC 8.8%, *P* = 0.68), intraoperative and postoperative complications (prolonged LC 5.7% and early LC 2.9%, *P* ≥ 0.99), operation time (prolonged LC 193.5 min and early LC 198.0 min, *P* = 0.81), and operation costs (prolonged LC 8,700 Yuan, and early LC 8,500 Yuan, *P* = 0.86). However, the prolonged LC was associated with longer postoperative hospitalization (7.0 days versus 6.0 days, *P* = 0.03), longer total hospital stay (11.0 days versus 8.0 days, *P* < 0.01), and subsequently higher total costs (40,400 Yuan versus 31,100 Yuan, *P* < 0.01).

**Conclusions:**

Prolonged LC is safe and feasible for patients with AC for having similar rates and severity of adverse events as early LC, but it is also associated with longer hospital stay and subsequently higher total cost.

Acute cholecystitis (AC) is a common acute abdominal disease for emergency admission and there is about 3–10% of acute abdominal pain linked to AC [1]. Right upper abdominal pain is the most typical symptom [2]. Usually a standardized treatment of laparoscopic cholecystectomy (LC) would be recommended for patients with AC [3–6]. Most researches[7–12] supported that LC should be done within 72 h of symptoms onset, which was nominated as early LC, due to the lower conversion rate, less intraoperative and postoperative complications, shorter hospitalization, and smaller amount of cost. According to TG18 (Tokyo Guideline 2018) [4], a delayed LC (i.e., performed at least 6 weeks after initial conservative treatment) might be proposed for patients with AC who had the onset of symptoms more than 72 h.

Actually, surgeons always encountered patients with symptoms as disgusting and recurrent abdominal pain lasting more than 72 h. These patients denied readmission for a delayed LC persistently. In addition, in clinical practice, there also may be a sudden attack forcing to an unprepared LC or even open cholecystectomy during the waiting for a delayed LC [4]. Furthermore, the conservative treatment and the following surgical hospitalization mean higher cost and more time consuming on the treatment of AC [13, 14]. Therefore, researches on the safety and feasibility of prolonged LC are needed. A previous randomized clinical trial analyzed prolonged and delayed LC and found that prolonged LC was safe and associated with less overall morbidity, shorter hospital stay, and less cost compared with delayed LC [15]. So, we would like to confirm the safety and feasibility of a prolonged LC conducted in patients with the onset of symptoms more than 72 h.

## Patients and methods

This study was approved by the ethics committee of Tongji medical college, Huazhong University of Science and Technology (Study Number 2019 S937).

### Patient selection

This single-center, retrospective study was performed at a department of emergency surgery in a tertiary academic medical center. During January 2015 and October 2018, patients diagnosed with AC and treated with LC at the same admission were included. The diagnosis of AC met the TG2018 [4], patients with at least one of local symptom or sign (murphy’s sign, right upper quadrant tenderness/pain/mass), one systemic sign (fever, evaluated C-reactive protein, evaluated white blood cell count (> 18,000 mm^3^)), and a confirmatory imaging test (color doppler ultrasound, computed tomography, and/or magnetic resonance cholangiopancreatography). The imaging evidences included the presence of gallstones, thickened gallbladder wall, pericholecystic fluid, and/or sonographic Murphy’s sign. All imaging examinations were carried out by trained radiologists. The cases with choledocholithiasis, pancreatitis, pregnancy, and patients under 18 years old were excluded.

### Basic characteristics

Data about age, sex, history of abdominal surgery, current smoking and drink, and pathological diagnosis were collected. The intraoperative pathological findings of simple, phlegmonous, or gangrenous cholecystitis were recorded. Each patient was assessed within the 24 h of admission by Acute Physiology and Chronic Health Evaluation IV (APACHE IV), Modified Early Warning Score(MEWS), Charlson Comorbidity Index (CCI), American Society of Anesthesiologists Physical Status Classification System (ASA-PS), Sequential Organ Failure Assessment(SOFA), quick Sequential Organ Failure Assessment (qSOFA), Systemic Inflammatory Response Syndrome(SIRS), Numerical Pain Rating Scale (NPRS), and the Barthel index of Activities of Daily Living (ADL).

### Operative outcomes

The primary outcomes were the mortality rate, admitted to intensive care unit (ICU) more than 24 h, conversion rate to open cholecystectomy (OC), intraoperative and postoperative complications including massive bleeding (> 500 ml) [16] during and/or after operation, wound infection, and biliary leakage. The secondary outcomes were the operation time, length of hospital stay, duration of postoperation hospitalization, surgical and total costs.

### Surgical procedure

All operations were performed by a fixed emergency surgeon team with 2–4 years of experience on laparoscopic techniques. LC was carried out with typical four incisions operation method [17, 18], and another incision maybe rarely needed in some difficult cases. LC conversion to OC was decided by the surgeon team independently.

### Statistical analysis

Data were analyzed with the Statistical Package for Social Science (SPSS), IBM, version 22.0. Continuous variables were described by mean (± standard deviation, SD), median (interquartile ranges, IQR), they were compared with Student’s *t* test and Mann–Whitney *U* test. Categorical variables were presented as percentages, they were compared with Pearson’s chi-square test and Fisher's exact test. *P* < 0.05 was considered significant.

## Results

**Fig. 1:**
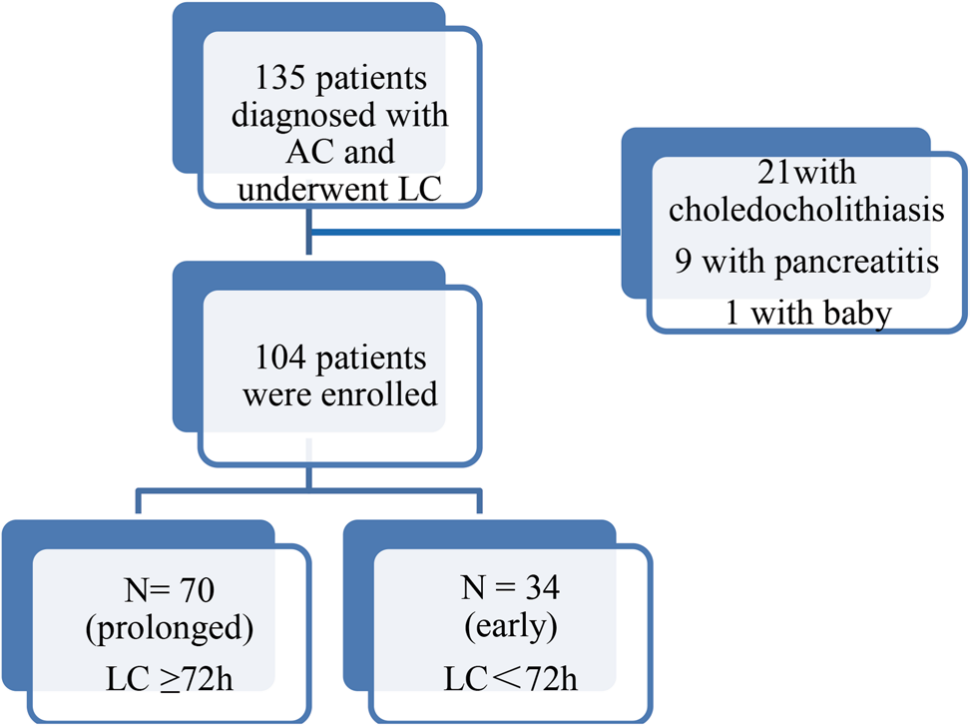
Flow diagram of patients enrolled

A total of 135 patients were admitted with the diagnosis of AC and underwent LC, 31 cases were excluded because of 21 cases with choledocholithiasis, 9 with acute pancreatitis, 1 with baby, there were 104 patients finally enrolled. Among the 104 patients 70 (67.3%) underwent prolonged LC, 34 (32.7%) underwent early LC (Fig. 1). The median time from symptoms onset to LC was 9 days with a range of 4–35 days and 3 days with a range of 1–3 days in the prolonged and early group, respectively.

**Fig. 2:**
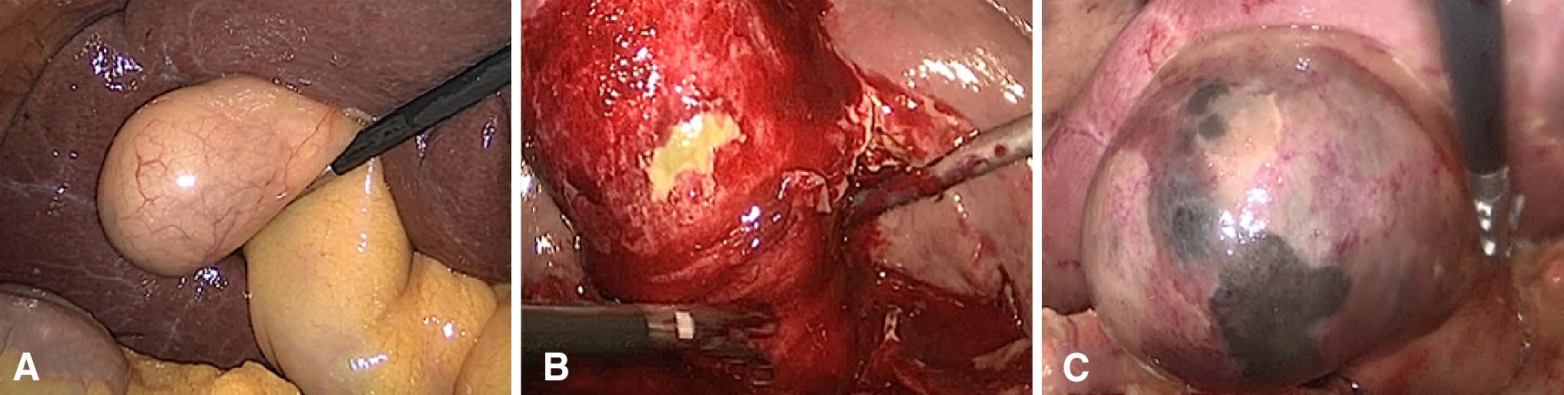
Clinical pathological diagnosis of acute cholecystitis. **A** Simple cholecystitis, **B** phlegmonous cholecystitis, and **C** gangrenous cholecystitis

Patients in prolonged and early groups had similar basic characteristics in age, gender, current drink and smoking, and history of abdominal surgery. There were no significant differences between the two groups on the assessment of APACHE IV, MEWS, CCI, ASA-PS, SOFA, qSOFA, SIRS, NPRS, and the Barthel index of ADL. In the prolonged group, patients presented with more serious classifications on TG18 (*P* < 0.01) though both groups had similar clinical (in surgery) and final pathological diagnosis (*P* = 0.95, *P* = 0.37) (Table 1). The overall morbidity of simple, phlegmonous, and gangrenous cholecystitis (Fig. 2) was 63.4%, 10.6%, and 36.0%. None died or admitted to intensive care unit (ICU) more than 24 h during the hospitalization. Both groups had similar conversion rate (prolonged LC 5.7%, and early LC 8.8%, *P* = 0.68). We found that 4 patients losing blood more than 500 ml during the operation in the prolonged group, and in the early group, 1 patient suffered from massive bleeding and wound infection. Though the prolonged group had higher intraoperative and postoperative complications than the early group (prolonged LC 5.7%, early LC 2.9%), there were no significant difference among them (*P* ≥ 0.99). The prolonged and early groups had similar operation time (prolonged LC 193.5 min, and early LC 198.0 min, *P* = 0.81) and surgery costs (prolonged LC 8700 Yuan, and early LC 8500 Yuan, *P* = 0.86), respectively. The total hospital stay and postoperation hospitalization were longer in the prolonged group (11.0 days versus 8.0 days, *P* < 0.01) and (7.0 days versus 6.0 days, *P* = 0.03). The prolonged group was also associated with higher total costs (40,400 Yuan versus 31,100 Yuan, *P* < 0.01) (Table 2).

**Table 1:** Baseline and clinical characteristics compared prolonged and early LC

Characteristics	Prolonged	Early	*P* value
	(*n* = 70)	(*n* = 34)	
Age (years) [mean ± SD]	54.4 (12.8)	50.0 (11.8)	0.42
Gender [*n* (%)]			0.16
Male	35 (50.0)	22 (64.7)	
Female	35 (50.0)	12 (35.3)	
Current drink [*n* (%)]	4 (5.7)	3 (8.8)	0.68
Current smoking [*n* (%)]	4 (5.7)	5 (14.7)	0.15
History of abdominal surgery [*n* (%)]	12 (17.1)	2 (5.9)	0.14
APACHE IV [ median (IQR)]	18.0 (14.0–24.5)	17.0 (7.8–22.0)	0.08
MEWS [ median (IQR)]	4.0 (3.0–5.0)	4.0 (4.0–5.0)	0.09
CCI [ median (IQR)]	3.0 (2.0–5.0)	2.5 (1.0–4.0)	0.13
ASA-PS [*n* (%)]			0.24
I	19 (27.1)	13(38.2)	
II	34 (48.6)	18(52.9)	
III	16 (22.9)	3 (8.8)	
IV	1 (1.4)	0 (0.0)	
SOFA score [ median (IQR)]	1.0 (0.0–2.0)	1.0 (0.0–1.0)	0.30
qSOFA scores [*n* (%)]			0.56
0	57(81.4)	36 (76.5)	
1	13 (18.6)	8 (23.5)	
SIRS criteria [*n* (%)]			0.7
0	23 (32.9)	7 (20.6)	
1	24(34.3)	12 (35.3)	
2	15 (21.4)	10 (29.4)	
3	5 (7.1)	3 (8.8)	
4	3 (4.3)	2 (5.9)	
NPRS [*n* (%)]			0.59
0	21 (30.0)	6 (17.6)	
1	11 (15.7)	7 (20.6)	
2	28 (40.0)	16 (47.1)	
3	10 (14.3)	5 (14.7)	
The Barthel index of ADL [*n* (%)]			0.15
50–70	24 (34.3)	6 (17.6)	
75–95	12 (17.1)	5 (14.7)	
100	34 (48.6)	23 (67.6)	
Severity grade of TG18 [*n* (%)]			**<** **0.01**
I (mild)	0 (0.0)	19 (55.9)	
II (moderate)	64 (91.4)	13 (38.2)	
III (severe)	6 (8.6)	2 (5.9)	
Clinical pathological diagnosis [*n* (%)]			0.95
Simple	45 (64.3)	21 (61.8)	
Phlegmonous	7 (10.0)	4 (11.8)	
Gangrenous	18 (25.7)	9 (26.5)	
Pathological diagnosis [*n* (%)]			0.37
Acute cholecystitis	25 (35.7)	17 (50.0)	
Acute on chronic cholecystitis	25 (35.7)	10 (29.4)	
Chronic cholecystitis	20 (28.6)	7 (20.6)	

*LC* Laparoscopic Cholecystectomy, *IQR* Inter Quartile Range, *APACHE IV* Acute Physiology and Chronic Health Evaluation IV, *MEWS* Modified Early Warning Score, *CCI* Charlson Comorbidity Index, *ASA-PS* American Society of Anesthesiologists Physical Status Classification, *SOFA* Sequential Organ Failure Assessment, *qSOFA* quick Sequential Organ Failure Assessment, *SIRS* Systemic Inflammatory Response Syndrome, *NPRS* Numerical Pain Rating Scale, *ADL* Activities of Daily Living, *TG18* Tokyo Guideline 2018

Bold values are statistically significant difference with alpha of 0.05

**Table 2:** Surgical characteristics and surgery-related outcomes

Characteristics and Outcomes	Prolonged	Early	*P* value
	(*n* = 70)	(*n* = 34)	
Mortality [*n* (%)]	0	0	–
ICU [*n* (%)]	0	0	–
Conversion rate [*n* (%)]	4 (5.7)	3 (8.8)	0.68
No. patients with complications [*n* (%)]	4 (5.7)	1 (2.9)	1.00
Bleeding (> 500 ml)	4	1	1.00
Wound infection	0	1	
Biliary leakage	0	0	
Operative time (min) [median (IQR)]	193.5 (150.0–247.5)	198.0 (150.0–266.3)	0.81
Duration of postoperation hospitalization (days) [median (IQR)]	7.0 (5.0–8.0)	6.0 (5.0–7.0)	**0.03**
Total hospital length of stay (days) [median (IQR)]	11.0 (8.0–14.0)	8.0 (6.0–9.0)	**< 0.01**
Cost for surgery (Yuan) [median (IQR)]	8700 (6500–12, 000)	8 500 (7100–11, 800)	0.86
Total cost of hospitalization (Yuan) [median (IQR)]	40, 400 (33, 900–51, 100)	31, 100 (24, 900–37, 000)	**< 0.01**

*ICU* intensive care unit, *IQR* inter quartile range

Bold values are statistically significant difference with alpha of 0.05

In Table 3, we analyzed the severe cholecystitis (grade III) which was classified on TG18. There were 6 (8.6%) severe cases in the prolonged group, 2 (2.9%) patients with renal dysfunction, 2 (2.9%) with hepatic dysfunction, and 2 (2.9%) with hematological dysfunction. And in the early group, 2 (5.9%) patients classified to the severe AC owing to hematological dysfunction.

**Table 3:** Evidences to patients classified to severe (grade III) cholecystitis on TG18

Evidences	Prolonged	Early
	(*n* = 70)	(*n* = 34)
Renal dysfunction	2	0
Hepatic dysfunction	2	0
Hematological dysfunction	2	2

*TG18* Tokyo Guideline 2018

**Fig. 3:**
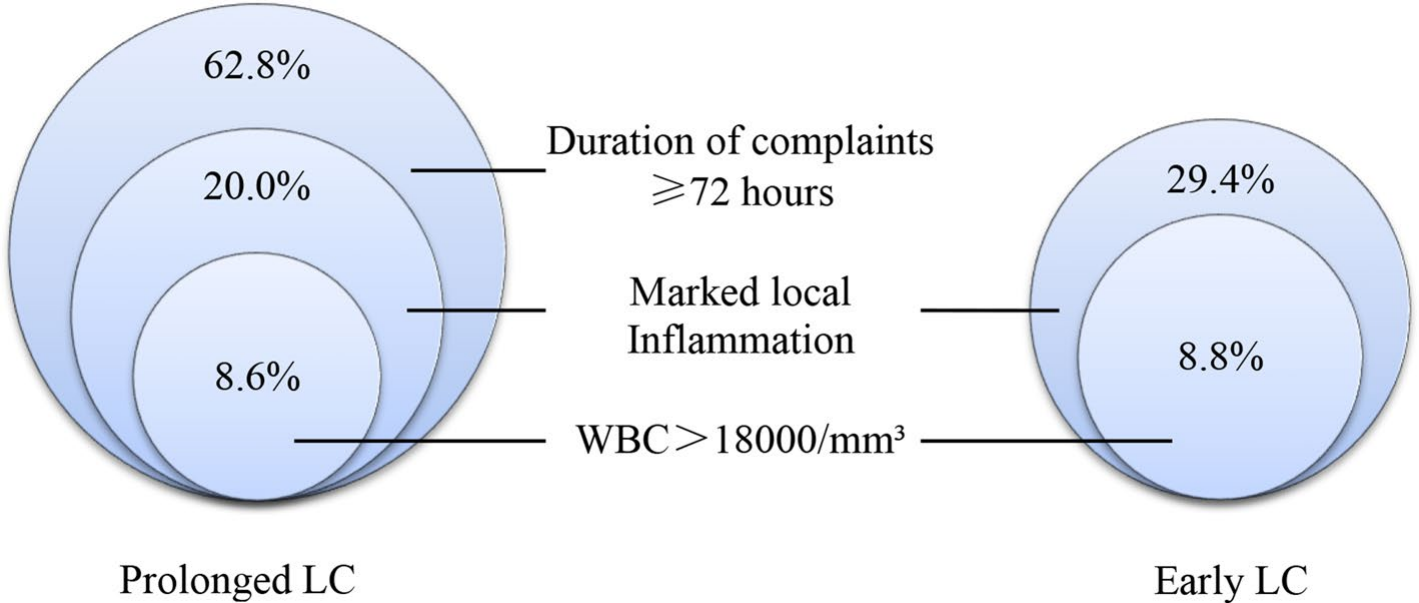
Evidences to patients classified to moderate cholecystitis in prolonged and early LC group on Tokyo Guideline 2018

In this study, patients classified to moderate cholecystitis in prolonged and early groups were 64 (91.4%) and 13 (38.2%), respectively. Figure 3 showed that there were 14 (20.0%) versus 10 (29.4%) patients classified to moderate (grade II) AC associated with marked local inflammation in the prolonged and early groups, and 6 (8.6%) versus 3 (8.8%) patients simultaneously suffered from elevated WBC (white blood cell count) and marked local inflammation, respectively. Then, we analyzed patients classified to moderate (grade II) AC due to the onset of symptoms being more than 72 h with the mild (grade I) ones and further verified the safety and feasibility of prolonged LC.

Table 4 showed that the moderate and mild groups had similar basic characteristics on age, gender, current drink and smoking, and history of abdominal surgery, proportional scores on APACHE IV, MEWS, CCI, ASA-PS, SOFA, qSOFA, SIRS, NPRS, and the Barthel index of ADL assessed within 24 h of admission, clinical and final pathological diagnosis. The conversion from LC to OC and intraoperative and postoperative complications were similar (1 vs 0 patient) and (1 vs 0 patient), respectively. There were no significant differences in moderate and mild groups on operation time (moderate 177.0 min, and mild 162.0 min, *P* = 0.66), duration of postoperation hospitalization (moderate 6.0 days, and mild 6.0 days, *P* = 0.26), and operation costs (prolonged 7,800 Yuan, and mild 8,100 Yuan, *P* = 0.72). The moderate group had longer total hospital stay (10.0 days versus 8.0 days, *P* < 0.01) and higher total costs (38,900 Yuan versus 30,000 Yuan, *P* < 0.01).

**Table 4:** Basic characteristics and outcomes compared moderate cholecystitis only with symptoms more than 72 h with mild cholecystitis

Characteristics	Grade II (moderate)	Grade I (mild)	*P* value
	(*n* = 44)	(*n* = 19)	
Age (years) [ mean ± SD]	53.4 (13.0)	49.3 (14.3)	0.27
Gender [*n* (%)]			0.11
Male	18 (40.9)	12 (63.2)	
Female	26 (59.1)	7 (36.8)	
Current drink [*n* (%)]	3 (6.8)	2 (10.5)	0.63
Current smoking [*n* (%)]	3 (6.8)	3 (15.8)	0.36
History of abdominal surgery [*n* (%)]	10 (22.7)	1 (5.3)	0.15
APACHE IV [ median (IQR)]	18.0 (14.0–22.5)	14.0 (4.0–21.5)	0.07
MEWS [ median (IQR)]	4.0 (3.0–5.0)	4.0 (4.0–4.5)	0.57
CCI [ median (IQR)]	3.0 (2.0–4.5)	2.0 (0.0–4.0)	0.21
ASA-PS [*n* (%)]			0.24
I	14 (31.8)	8 (42.1)	
II	18 (40.9)	10 (52.6)	
III	11 (25.0)	1 (5.3)	
IV	1 (2.3)	0 (0.0)	
SOFA score [ median (IQR)]	0.0 (0.0–2.0)	0.0 (0.0–1.0)	0.25
qSOFA scores [*n* (%)]			0.49
0	37 (84.1)	14 (73.7)	
1	7 (15.9)	5 (26.3)	
SIRS criteria [*n* (%)]			0.39
0	17 (38.6)	4 (21.1)	
1	17 (38.6)	9 (47.4)	
2	8 (18.2)	4 (21.1)	
3	1 (2.3)	2 (10.5)	
4	1 (2.3)	0 (0.0)	
NPRS [*n* (%)]			0.27
0	14 (31.8)	2 (10.5)	
1	10 (22.7)	4 (21.1)	
2	14 (31.8)	10 (52.6)	
3	6 (13.6)	3 (15.8)	
The Barthel index of ADL [*n* (%)]			0.57
50–70	15 (34.1)	4 (21.1)	
75–95	5 (11.4)	3 (15.8)	
100	24 (54.5)	12 (63.2)	
Severity grade of TG18 [*n* (%)]			0.46
I (mild)	24 (54.5)	14 (73.3)	
II (moderate)	8 (18.2)	2 (10.5)	
III (severe)	12 (27.3)	3 (15.8)	
Clinical pathological diagnosis [*n* (%)]			0.27
Simple	53.4 (13.0)	49.3 (14.3)	
Phlegmonous	18 (40.9)	12 (63.2)	
Gangrenous	26 (59.1)	7 (36.8)	
Pathological diagnosis [*n* (%)]			0.06
Acute cholecystitis	5 (11.4)	7 (36.8)	
Acute on chronic cholecystitis	21 (47.7)	6 (31.6)	
Chronic cholecystitis	18 (40.9)	6 (31.6)	
Mortality [*n* (%)]	0	0	–
ICU [*n* (%)]	0	0	–
Conversion rate [*n* (%)]	1(2.3)	0(0.0)	–
No. patients with complications [*n* (%)]	1(2.3)	0(0.0)	–
Operative time (min) [median (IQR)]	177.0 (133.0–220.0)	162.0 (125.0–208.5)	0.66
Duration of postoperation hospitalization (days) [median (IQR)]	6.0 (5.0–8.0)	6.0 (4.5–6.5)	0.26
Total hospital length of stay (days) [median (IQR)]	10.0 (8.0–13.0)	8.0 (5.5–9.0)	< **0.01**
Cost for surgery (Yuan) [median (IQR)]	7800 (5900–11, 000)	8100 (6700–9400)	0.72
Total cost of hospitalization (Yuan) [median (IQR)]	38, 900 (32, 500–45, 500)	30, 000 (24, 300–35, 400)	< **0.01**

*LC* Laparoscopic Cholecystectomy, *IQR* Inter Quartile Range, *APACHE IV* Acute Physiology and Chronic Health Evaluation IV, *MEWS* Modified Early Warning Score, *CCI* Charlson Comorbidity Index, *ASA-PS* American Society of Anesthesiologists Physical Status Classification, *SOFA* Sequential Organ Failure Assessment, *qSOFA* quick Sequential Organ Failure Assessment, *SIRS* Systemic Inflammatory Response Syndrome, *NPRS* Numerical Pain Rating Scale, *ADL* Activities of Daily Living, *TG18* Tokyo Guideline 2018, *ICU* intensive care unit

Bold values are statistically significant difference with alpha of 0.05

## Discussion

This retrospective study based on real clinical problem and confirmed the safety and feasibility of prolonged LC compared to early LC. Prolonged LC had not increased the conversion rate, intraoperative and postoperative complications, operation time, and cost. However, prolonged LC was associated with longer postoperation and total hospital stay and higher total cost compared to early LC. Our study indicated that early LC was superior to prolonged LC for patients with AC. It is particularly worth mentioning here that higher rate of prolonged LC was observed in our study (67.3% VS 32.7%), although early LC was recommended in clinical practice. We reviewed the medical records and found that most of the patients missed the golden 72 h when they went to see a doctor, and a few of them waited for the final diagnosis of AC. According to our results, for these patients, prolonged LC was recommended, rather than conservative treatment and waiting for a delayed LC.

There were many studies on the optimal time of surgery for AC. Numerous of evidence indicated that early LC is the first choice and secure when the duration of symptoms was less than 72 h [4, 8, 19, 20]. And for those more than 72 h, timely LC was better than delayed LC [15]. Another large, multicenter, prospective randomized clinical trial [21] compared early LC within 24 h of symptoms with delayed LC in 7–45 days of presentation, and confirmed that early LC should be performed for patients with AC. Thus, our study was complementary to these researches, for we defined the prolonged LC as patients with AC had LC beyond 72 h of symptoms onset and confirmed the safety and feasibility of prolonged LC for AC.

**Fig. 4:**
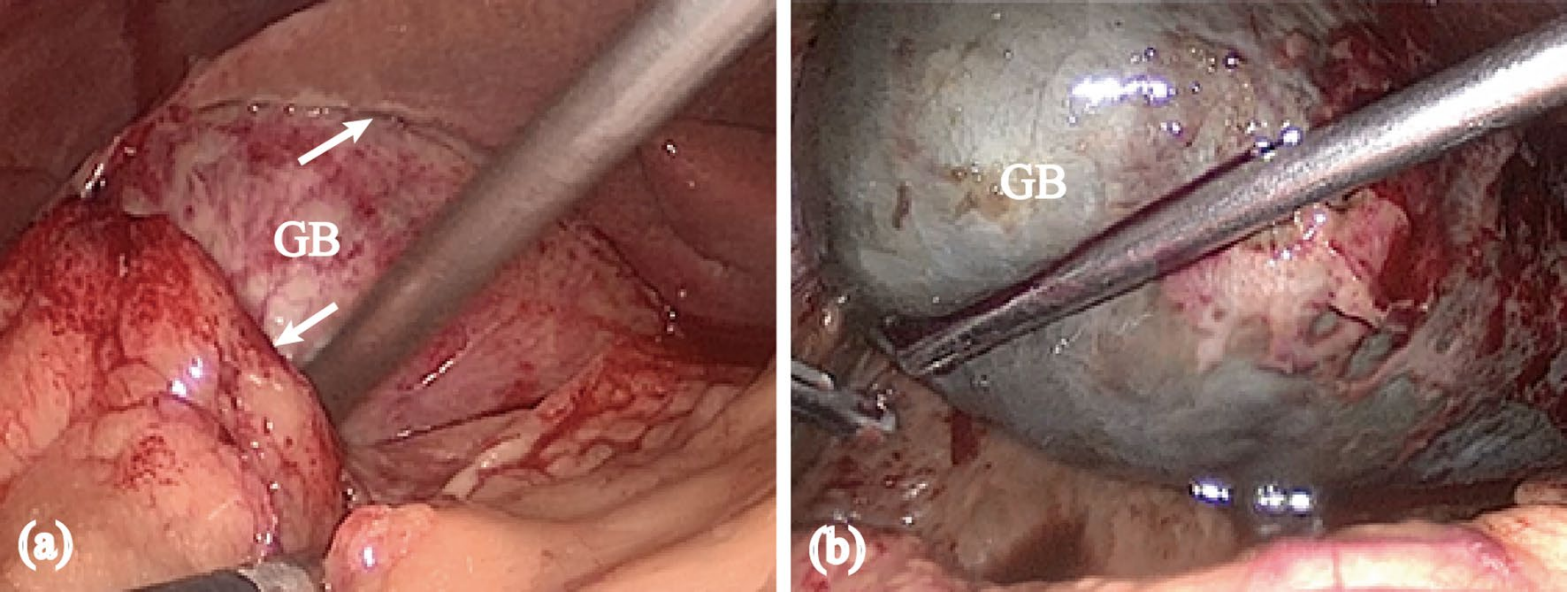
Difficult cases in clinical practice. **A** The gallbladder (GB) was tightly adherent with liver and omentum (arrows). **B** Severe gangrenous GB, which was black and distended

Several studies [21, 22] have shown that early LC is superior to the delayed LC, but another real-world clinical study [23] indicated that a delayed LC is safe, and has better outcomes in morbidity and mortality. The opposite conclusions were probably derived from discrepant severity compositions of patients. In our study, we adopted several scoring systems to assess and standardize the responses of patients induced by the acute local or systemic inflammation at their admissions. The prolonged and early groups had equal scoring levels in each scoring system and it ensured the comparable preoperative states. Furthermore, both groups had similar clinical and final pathological diagnosis ranging from simple, phlegmonous, and gangrenous cholecystitis. There were also some really difficult cases with severe gangrene or tight adhesion to surrounding tissues (Fig. 4), demonstrating that we included not just the ‘easy’ cases in our study. Therefore, the cases we enrolled in our study were good representation for the research target.

In this retrospective analysis, the overall morbidity of gangrenous cholecystitis (GC) was 27/104 (26.0%), which was relatively high when compared to the reported morbidity of 5–26% for GC [24–28], indicating that the patients with AC were really serious in our emergency department, which may also contribute to the longer overall operation time than that reported previously (195.0 min vs 88 min-116 min)[15, 29].

There were 8 patients classified to severe cholecystitis, 6 patients underwent prolonged LC, and 2 underwent early LC (Table 3). All of them recovered well at discharge. On the contrary, conservative management before a delayed LC was successful in only 60.6% of cases, 14.7% of patients required emergency surgery due to gangrene and/or perforation [30]. It indicated the removal of inflamed gallbladder as soon as possible might be preferable than a conservative intervention. Therefore, in some severe cases (grade III) with renal, hepatic, or hematological dysfunction, an aggressive early LC or prolonged LC could be performed with carefully assessment before the operation rather than a delayed LC.

There were similar percent of evidences (marked local inflammation, evaluated WBC) contributing to the classified to grade II (moderate) cholecystitis in the prolonged and early groups (Fig. 2), which indicated that evaluated WBC was concomitant to the local infection. And it might imply that the systemic immunological reactions resulted from the local inflammation in this study.

A limitation of this study was that patients were retrospectively admitted from emergency surgery department in a single center, and further prospective researches in multicenter were needed to be performed.

## Conclusion

Prolonged LC for patients with AC was safe and feasible, and when patients with AC missed the golden 72 h for LC, a prolonged LC could be recommended for them.
